# Topical insulin used alone or in combination with drug-depository contact lens for refractory cases of neurotrophic keratopathy

**DOI:** 10.1016/j.ajoc.2024.102227

**Published:** 2024-11-28

**Authors:** Alessandra Mancini, Maura Mancini, Andrea Taloni, Luca Bifezzi, Maria Angela Romeo, Lorenzo Rijillo, Mario Verdiglione, Vincenzo Scorcia, Pasquale Aragona, Giuseppe Giannaccare

**Affiliations:** a Department of Ophthalmology, University Magna Graecia of Catanzaro, Catanzaro, 88100, Italy; b Department of Biomedical Sciences, Ophthalmology Clinic, University of Messina, Messina, 98122, Italy; c Compounding Pharmacy, Farmacia Europea, Catanzaro, 88100, Italy; d Eye Clinic, Department of Surgical Sciences, University of Cagliari, Cagliari, 09124, Italy

**Keywords:** Insulin eye drops, Hyper-CL, Neurotrophic keratopathy, NK, Persistent epithelial defect

## Abstract

**Purpose:**

To report the clinical outcomes achieved in refractory cases of neurotrophic keratopathy (NK) through the utilization of insulin eye drops alone or in conjunction with a drug-depository contact lens (DDCL).

**Observations:**

This multicentric prospective open-label uncontrolled case series included consecutive patients with NK refractory to conventional treatment. Insulin eye drops (1 unit/mL) were prescribed 4 times/day in all cases, and a Therapeutic Hyper-CL™ soft contact lens (EyeYon Medical, Ness Ziona, Israel), designed to act as a drug reservoir, was applied in selected patients. Data about stage and duration of NK, corneal sensitivity, previous treatments, rate and speed of healing, changes of NK area over time were collected. Nine eyes of 8 patients (mean age 52.50 ± 12.03 years [95 % CI, 44.13–60.87]) affected by NK refractory to conventional medical therapy were included. All patients received topical insulin, while DDCL was also applied in 3 cases. At T0, the mean area of the corneal epithelial defect was 21.84 ± 18.35 mm^2^ [95 % CI, 9.86–33.84]. Complete corneal re-epithelialization occurred in all cases, after a mean time interval of 25.78 ± 8.39 days [95 % CI, 20.30–31.26]. Mean reduction rate of epithelial defect areas was −0.81 ± 0.44 mm^2^/day [95 % CI, −1.16 to −0.46] for patients treated with insulin eye drops, and −0.63 ± 0.30 mm^2^/day [95 % CI, −0.96 to −0.29] for those treated with insulin eye drops plus DDCL (p = 0.71). Neither adverse events nor episodes of NK recurrence were reported.

**Conclusions and importance:**

Topical insulin, used alone or in combination with DDCL, is an accessible, inexpensive, and effective treatment for refractory NK.

## 1 Introduction

The cornea is an essential component of the ocular dioptric system and plays a crucial role as a protective barrier against infectious agents through tight junctions between cells. Additionally, it maintains a smooth optical surface by constantly regenerating cells in the basal layer.^1^^,^^2^ As the most exposed layer of the eye, the cornea is susceptible to common ocular pathologies such as abrasions and epithelial defects, which are among the most fearsome conditions encountered in ophthalmic clinical practice. Persistent epithelial defects (PEDs) can result from altered epithelial adhesion, limbal stem cell deficiency, neurotrophic keratopathy (NK), physical injury, eyelid abnormalities, idiopathic and hereditary conditions, medications and infections. In fact, all these conditions can disrupt the physiological corneal healing process.^3^, ^4^, ^5^

Among them, NK is a corneal degenerative disease characterized by a reduction or absence of corneal sensitivity. Washout of epitheliotoxic drugs and lubrication with preservative-free artificial tears and ointments, often in combination with the use of bandage contact lens (CL), are the first line of NK treatment. Autologous and allogeneic blood-derived products have been described as effective second-line treatments.^6^, ^7^, ^8^ Surgical treatments have limited efficacy and include tarsorrhaphy, amniotic membrane transplantation and tectonic keratoplasty, while the recently described corneal neurotization is the only surgical technique that allows the improvement of corneal sensation and epithelial healing.^9^

Recently, several new medical treatments, either already available or under development, have been proposed for NK. These include topical fibronectin, thymosin beta 4, recombinant human nerve growth factor (rhNGF) (Cenegermin, Oxervate, Dompè, Italy), topical epidermal growth factor, human growth hormone, eye drops containing albumin or amniotic membrane extract, insulin-like growth factor (IGF)-1, and topical insulin 10,11. Insulin-like growth factors interact with insulin and IGF receptors, playing a crucial role in the growth, differentiation, and proliferation of corneal epithelial cells. In fact, it has been demonstrated that insulin improves corneal epithelial cell proliferation and wound healing.^12^, ^13^, ^14^, ^15^, ^16^, ^17^ Topical insulin offers several advantages, including safety, tolerability, easy availability, along with a favorable cost-effectiveness ratio.

The purpose of this study is to present the outcomes of refractory cases of NK treated with topical insulin; in selected cases of the cohort, a soft CL designed to act as a drug reservoir was applied aiming at increasing the bioavailability of the insulin on the corneal surface.^18^

## 2 Materials and Methods

In this prospective, open-label, uncontrolled case series conducted at three university centers in Italy (i) “Magna Graecia” University of Catanzaro; ii) University of Cagliari; iii) University of Messina) consecutive patients with refractory NK were included. Insulin eye drops (1 unit per mL) were prepared by a compounding pharmacy (Farmacia Europea, Catanzaro, Italy) in a grade D cleanroom and prescribed 4 times daily until the closure of the epithelial defect. In selected patients with a previous history of bandage CL wearing who did not present any contraindication (tarsorrhaphy, floppy eyelid syndrome or symblepharon), insulin therapy was combined with the Therapeutic Hyper-CL™ soft CL (EyeYon Medical, Ness Ziona, Israel). This CL was designed to act as a drug depot, with the aim of increasing the bioavailability of the insulin eye drops on the corneal surface. The study adhered to the ethical guidelines of the Declaration of Helsinki. Risks, benefits, and alternative treatment options were discussed with all patients, and informed consent was obtained for the off-label use of insulin and drug-depository CL (DDCL). Inclusion criteria included patients with NK persisting for more than 4 weeks who did not respond to ongoing conventional treatment (e.g. preservative-free artificial tears and ointments, anti-inflammatory therapy, bandage CL, punctal plugs, and autologous serum). Study variables included: patient age and sex, etiology, stage (according to Mackie classification) and duration (interval from NK diagnosis to initiation of study treatment [T0]) of NK; area of the epithelial defect measured in mm^2^ using image analysis with ImageJ software 1.53t (National Institutes of Health, Bethesda, MD; available at: http://imagej.nih.gov/ij); corneal sensitivity using the Cochet-Bonnet esthesiometer; previous and concomitant treatments; time and speed of healing; complications or side effects. Patients were evaluated at T0 and every 7 ± 2 days until complete healing. After NK resolution, patients were followed-up monthly to monitor the risk of NK recurrence.

Values were expressed as mean ± standard deviation. The reduction rates of NK area were assessed over time, and the Mann-Whitney *U* test was used to evaluate the difference in reduction rates between patients treated with insulin alone and those treated with insulin plus DDCL. A p value < 0.05 was considered significant.

## 3 Results

**Fig. 1:**
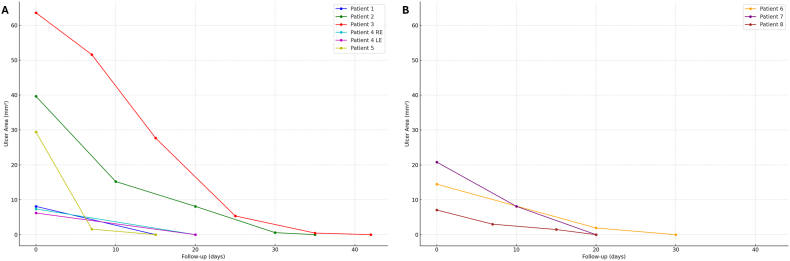
**Graph illustrating the changes over time of the epithelial defect.** Areas of epithelial defect evaluated over time in patients with neurotrophic keratopathy treated with insulin alone (a) or in combination with a drug-depository contact lens (b).

Nine eyes of 8 patients (4 M, 4 F, mean age 52.50 ± 12.07 years) affected by NK (stage II in 8 cases and stage III in one case) refractory to conventional medical therapy were included. The mean NK duration was 47.37 ± 26.86 days [95 % CI, 28.76–65.98]. All eyes received topical insulin, while DDCL was applied in 3 cases (patients #6, #7, #8). At T0, the mean area of the corneal epithelial defect was 21.84 ± 18.35 mm^2^ [95 % CI, 9.86–33.84] and corneal sensitivity was absent in 8 out of 9 cases. Complete corneal re-epithelialization occurred in 9 out of 9 cases (100 %) after a mean time interval of 25.78 ± 8.39 days [95 % CI, 20.30–31.26]. Fig. 1 shows the reduction over time of epithelial defect areas in patients treated with insulin eye drops alone (part A) or insulin eye drops plus DDCL (part B). Overall, the mean reduction rate of epithelial defect areas was −0.75 ± 0.41 mm^2^/day [95 % CI, −1.01 to −0.48]. Mean reduction rate of epithelial defect areas was −0.81 ± 0.44 mm^2^/day [95 % CI, −1.16 to −0.46] for patients treated with insulin eye drops, and −0.63 ± 0.30 mm^2^/day [95 % CI, −0.96 to −0.29] for those treated with insulin eye drops plus DDCL. No statistically significant difference in reduction rates was found between the two groups of patients (p = 0.71). No adverse events were recorded during the study period. Patients were followed-up for at least 3 months after complete closure of the epithelium with no episode of NK recurrence.

**Fig. 2:**
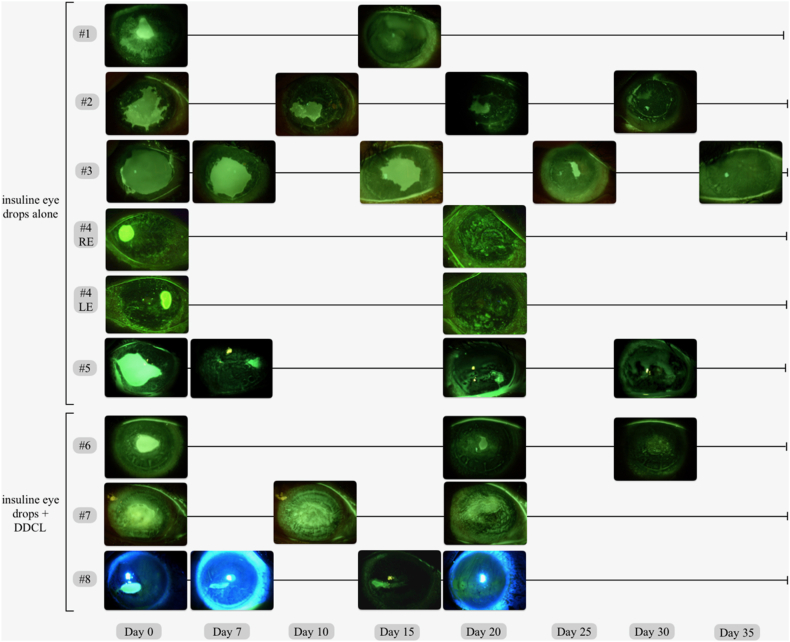
**Slit lamp photographs of all patients before and during therapy.** Slit lamp photographs of the cornea with yellow/blue filter collected at T0 and during subsequent time points until complete healing. (For interpretation of the references to colour in this figure legend, the reader is referred to the Web version of this article.)

Table 1 summarizes demographical and clinical characteristics of patients enrolled in the study along with the outcomes of the study treatment. Fig. 2 shows slit lamp photograph of NK over time for all patients at each time point.

**Table 1:** Demographical and clinical data of patients with refractory neurotrophic keratopathy treated with insulin eye drops used alone or in combination with a drug-depository contact lens (DDCL). Abbreviations: NK = neurotrophic keratopathy; cn = cranial nerve; RE = right eye; LE = left eye; OU = both eyes; CL = contact lens; M = male; F = female; CN = cranial nerve; PK = penetrating keratoplasty; DALK = deep anterior lamellar keratoplasty.

ID	Diagnosis	Sex	Age (y)	Side	NK stage	NK duration (from onset to initiation of study therapy) (days)	Corneal sensitivity at T0 (mm)	Area of epithelial defects at T0 (mm^2^)	Previous treatments	Combination with DDCL	Complete healing (time in days)	Complications/side effects
1	NK secondary to V cn palsy	M	58	RE	II	60	0	8.097	Preservative-free tear substitutes, Vitamin A ointment, autologous serum eye drop, punctual plugs	No (Floppy eyelid syndrome)	Yes (15)	No
2	NK after failed PK	M	65	RE	II	30	0	39.645	Preservative-free tear substitutes, autologous serum eye drop	No (Symblepharon)	Yes (35)	No
3	NK secondary to V cn palsy	F	42	RE	II	30	0	63.569	Preservative-free tear substitutes, Hydrocortisone Sodium Phosphate eye drop, autologous serum eye drop	No (No previous bandage contact leans wearing)	Yes (42)	No
4	NK secondary to dry eye owing to Sjogren's Syndrome	F	63	OU	II	30	RE 0 LE 0	RE 7.357 LE 6.198	Warm compresses, preservative-free tear substitutes, cyclosporine A 0.1 %, autologous serum eye drops	No (No previous bandage contact leans wearing)	Yes (20)	No
5	NK secondary to V and VII cn palsies	M	70	RE	II	21	0	29.423	Preservative-free tear substitutes, Vitamin A ointment, nocturnal patch, lateral tarsorraphy	No (Tarsorrhaphy)	Yes (30)	No
6	Post-herpetic NK in DALK graft	F	37	LE	II	90	10	14.477	Preservative-free tear substitutes, Hydrocortisone Sodium Phosphate eye drop, bandage CL, autologous serum eye drop, acyclovir 800 mg tablets	Yes	Yes (30)	No
7	NK secondary to V cn palsy	M	40	RE	III	90	0	20.802	Preservative-free tear substitutes, Vitamin A ointment, bandage CL, autologous serum eye drop, fluorometholone, punctual plugs	Yes	Yes (20)	No
8	NK secondary to V and VII cn palsies	F	45	RE	II	28	0	7.080	Preservative-free tear substitutes, Vitamin A ointment, bandage CL, topical antibiotics	Yes	Yes (20)	No

### 3.1 Patient #1

A 58-year-old man, diagnosed with NK secondary to trigeminal neuralgia treated with alcoholization approximately 4 years before, presented at our center with a NK developed in the previous 2 months in the right eye (RE). Initial corneal neovascularization was observed, with no signs of active infection. Esthesiometry using the Cochet-Bonnet revealed the absence of corneal sensitivity in all the quadrants of the affected eye. Fluorescein staining revealed a central area of epithelial defect of 8.097 mm^2^ (width of 3.15 mm and height of 3.59 mm). A marked laxity of the upper and lower eyelids (floppy eyelid syndrome) was also present. The clinical picture was stable in the last month with no signs of improvement despite the ongoing therapy including preservative-free artificial tears, vitamin A ointment, punctal plugs, and autologous serum eye drops applied every 2 hours. Insulin eye drops were prescribed at a dosage of one drop 4 times a day, and the remaining therapy was stopped. After 2 weeks of treatment, the corneal epithelium completely healed with a slight residual stromal opacity. No recurrences or adverse events were noted until the last follow-up visit occurring 3 months later.

### 3.2 Patient #2

A 65-year-old man presented with NK following penetrating keratoplasty (PK) in the RE. The patient had a history of corneal perforation due to severe dry eye and blepharitis, treated in the acute phase with a conjunctival autograft according to Gundersen technique followed by optical PK. Slit-lamp examination revealed interrupted stitches still in place and NK with an epithelial defect involving an area of 39.645 mm^2^ (width of 7.33 mm and height of 8.45 mm). Corneal sensitivity was completely absent. Symblepharon was also present in the inferior fornix. Previous medical therapy included preservative-free artificial tears instilled 8 times a day, vitamin A ointment, and autologous serum eye drops every 2 hours. Given the lack of improvement despite 1 month of therapy, insulin eye drops were prescribed at a dosage of one drop 4 times a day and the other therapies were discontinued. After 3 weeks of treatment, the area of the epithelial defect had significantly reduced to 8.095 mm^2^ (width of 3.601 mm and height of 2.380 mm). After 2 further weeks of treatment, the epithelial defect completely resolved. Currently, 2 months after the complete NK healing, the epithelium continues to be integrum.

### 3.3 Patient #3

**Fig. 3:**
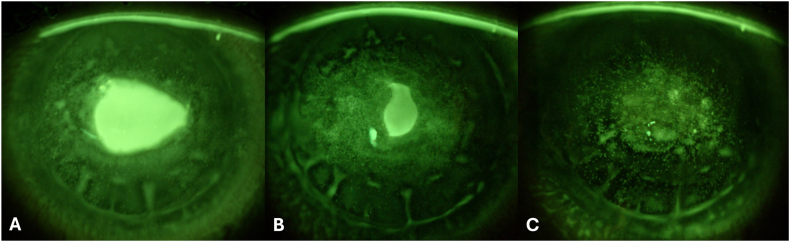
**Slit lamp photographs of patient #6 before and during therapy.** Slit lamp photographs of the cornea with yellow/blue filter collected at T0 (a), day 20 (b), and day 30 (c). (For interpretation of the references to colour in this figure legend, the reader is referred to the Web version of this article.)

A 42-year-old woman with a history of microvascular decompression of the trigeminal nerve presented with a NK in the RE involving an area of 63.569 mm^2^ (width of 9.76 mm and height of 8.27 mm). At the time of presentation, the patient was using preservative-free artificial tears, hydrocortisone sodium phosphate twice a day, and autologous serum eye drops every 2 hours. Corneal esthesiometry showed a complete lack of sensitivity in all the regions. Insulin eye drops were prescribed at a dosage of one drop 4 times a day and the remaining therapy was stopped. After 2 weeks of treatment, the defect area reduced to 27.693 mm^2^ (width of 6.45 mm and height of 5.93 mm). After 6 weeks of treatment, the epithelial defect completely resolved (Fig. 3). Currently, 6 months after the initial presentation, the patient shows no signs of disease recurrency.

### 3.4 Patient #4

A 63-year-old woman with secondary Sjogren's syndrome presented with NK in both eyes. Her clinical history included undifferentiated connective tissue disease, Hashimoto's thyroiditis, and scleroderma. She had been on systemic therapy with hydroxychloroquine 200 mg/day for 20 years. Clinical evaluation revealed reduced tear film stability, conjunctival epithelial alterations with folds, mucous plaques in the tear film, conjunctivalization at the sclero-corneal limbus, and NK involving the paracentral region of the cornea. Corneal sensitivity was absent in both eyes. The initially prescribed therapy in both eyes included warm compresses, preservative-free tear substitutes containing 0.2 % sodium hyaluronate, 0.1 % cyclosporine A eye drops administered 4 times a day, and autologous serum eye drops administered 6 times a day. Given the lack of improvement despite 1 month of treatment, insulin eye drops were prescribed at a dosage of one drop 4 times a day and the remaining therapy was stopped. At T0, NK area was 7357 mm^2^ in the RE (width of 3.027 mm and height of 3.093 mm), and 6.198 mm^2^ in the left eye (LE) (width of 2.202 mm and height of 3.554 mm). After 3 weeks of this modified treatment regimen, complete closure of the epithelial defect was reached. At the 6-month follow-up, the epithelium remained intact without signs of recurrence.

### 3.5 Patient #5

A 70-year-old man with a history of V and VII cranial nerves palsies presented in the LE a clinical picture of NK involving an area of 29.423 mm^2^ (width of 8.54 mm and height of 6.09 mm). A partial tarsorrhaphy had been previously performed to limit the ocular surface exposure. Ongoing medical therapy, which determined no improvement over 3 weeks, included preservative-free artificial tears, vitamin A ointment, and nocturnal patch. Insulin eye drops were prescribed at a dosage of one drop 4 times a day and the remaining therapy was stopped. After 1 week of treatment, the epithelial defect reduced to an area of 1.573 mm^2^ (width of 1.47 mm and height of 1.40 mm), reaching a complete healing after further 3 weeks of treatment. Upon discontinuation of insulin eye drops for the subsequent 3 months, the course of the patients was regular without any recurrence.

### 3.6 Patient #6

A 37-year-old woman came to our attention with a previous history of deep anterior lamellar keratoplasty (DALK) in the LE for keratoconus (2017) and subsequent repeated DALK (2019) due to opacity of the DALK graft following an episode of herpetic keratitis. After the second DALK procedure, the graft remained clear and epithelialized for 3 years until when the patient presented to our center with a stage II NK. There was a severe corneal hypoesthesia recorded with the Cochet-Bonnet esthesiometer (10 mm). After 3 months of treatment without improvement, which included daily preservative-free artificial tears, bandage CL, and autologous serum eye drops every 2 hours, insulin eye drops were prescribed at a dosage of one drop 4 times a day, the remaining therapies were stopped and DDCL was applied. At T0, the area of the epithelial defect of 14.477 mm^2^ (width of 4.90 mm and height of 3.78 mm). The 3-week follow-up visit showed a gradual reduction in the size of the NK to an area of 1.933 mm^2^ (width of 1.31 mm and height of 2.08 mm). One week later, a complete healing was reached with residual punctate epithelial erosions and a central leukoma. No adverse events were reported and after the last follow-up visit scheduled 3 months after the complete healing the picture was stable.

### 3.7 Patient #7

A 40-year-old man with a 3-month history of NK developed after surgical and radiotherapeutic treatment for an extensive nasosinusal carcinoma extending to the skull base, presented with a central epithelial defect in the RE. The outcomes of this case have been already reported by us.^18^ Briefly, slit-lamp examination revealed conjunctival hyperemia, deep corneal neovascularization, keratolysis and corneal thinning. The corneal epithelial defect measured an area of 20.802 mm^2^ (width of 6.06 mm and height of 4.09 mm). Complete corneal anesthesia was also found. Despite 3 months of treatment with preservative-free artificial tears, vitamin A ointment, punctal plugs, bandage CL, and autologous serum eye drops every 2 hours, no improvements were observed. Consequently, insulin eye drops were prescribed at a dosage of one drop 4 times a day, the remaining therapies were stopped and DDCL was applied to extend the interaction between the eye drops and the corneal surface. During the first visit, a reduction in the epithelial defect was observed, while a complete healing was obtained after 3 weeks when also a reduction in conjunctival hyperemia and corneal opacity was noted. No adverse events were reported. Currently, 6 months after the complete healing the patient uses preservative-free artificial tears, and the corneal epithelium remains closed.

### 3.8 Patient #8

A 45-year-old woman with a history of surgical intervention for an acoustic neuroma using a trans-labyrinthine approach causing V and VII cranial nerves palsies, presented to our attention with a stage II NK in the RE. Corneal sensitivity was absent in all regions. The prior ineffective medical therapy, which included preservative-free artificial tears administered multiple times a day, vitamin A ointment, and topical antibiotics 4 times a day, was interrupted and after a washout of further 2 weeks with no improvement, insulin eye drops in a galenic formulation (1 unit per mL), were prescribed 4 times a day, in combination with DDCL wearing. At T0, the epithelial defect measured an area of 7.080 mm^2^ (width of 4.07 mm and height of 2.01 mm). After 7 days of treatment, the area of the epithelial defect reduced to 3.014 mm^2^ (width of 3.99 mm and height of 0.93 mm); after further 2 weeks of treatment, a complete healing of NK was reached. Currently, at the 3-month follow-up, the epithelium is integrum without signs of recurrence.

## 4 Discussion

In the present case series, the positive outcomes of 9 eyes of 8 patients with refractory NK treated with insulin eye drops as the only therapy (n = 6) or in combination with DDCL wearing (n = 3) were reported. All patients achieved complete healing and did not show any sign of disease recurrency over the subsequent follow-up period of at least 3 months. These results are noteworthy, because the clinical course of NK can be challenging with sight-threatening complications such as keratolysis and corneal perforation. A wide armamentarium of treatments is available for the management of NK. First-line options include washout of epitheliotoxic products in use, preservative-free artificial tears, ointment and bandage CL. In case of disease persistence, serum or other blood derivatives eye drops can be introduced,^7^^,^^8^ while tarsorrhaphy and amniotic membrane grafting are usually performed to reduce the exposure of the eye and to provide a mechanical support for wound healing, respectively. In the last years, there has been a growing interest in the use of growth factors, and in particular rhNGF, whose use has been approved in this setting in 2017 (Europe) and 2018 (United States), providing good results in terms of NK healing (69.6 %).^11^ However, the high cost of the suggested 8-week treatment period is limiting its widespread adoption in the routine clinical practice. Another recent advancement is represented by the surgical procedure called corneal neurotization, which addresses the root of the pathology by restoring sensory function, protecting against recurrent ulceration, and improving visual acuity.^9^^,^^19^, ^20^, ^21^

Topical insulin promotes corneal re-epithelialization, showing a high profile of safety and tolerability, with a favorable cost-effectiveness ratio.^12^ The benefit of insulin is believed to derive from the regeneration of corneal nerves and/or stimulation of migration and proliferation of limbal epithelial cells.^14^^,^^15^ Furthermore, IGF-1 stimulates the expression of IGF receptors in limbal cells, promoting the differentiation of limbal stem cells. Its corneal effect has been studied in rodent models, particularly in diabetic rats, where Zagon et al. demonstrated that insulin improves corneal sensitivity, promotes wound healing after corneal abrasions, and slows the loss of corneal nerves in the sub-basal plexus.^22^^,^^23^ Topical insulin is an easily administered treatment, highly available and accessible, characterized by a favorable cost-effectiveness ratio, excellent tolerability and efficacy, without adverse effects. In a recent randomized clinical trial versus artificial tears, it was shown to be more effective for the healing of postoperative corneal epithelial defects induced during vitreoretinal surgery in diabetic patients.^24^

In our cohort present herein, insulin eye drops were used in 3 out 9 eyes in combination with DDCL, aiming at increasing the topical drug residence time. DDCL was applied in NK patients who were previously using a standard bandage CL (patients #6, #7 and #8), while was avoided in cases complicated by floppy eyelid syndrome (patient #1), symblepharon (patient #2) and partial tarsorrhaphy (patient #5), as these conditions usually hamper the correct wearing of a large-diameter (15.5 mm) CL. No significant differences were found when analyzing separately the outcomes of patients who received insulin as the only therapy compared to those who wore also DDCL. The lack of significance may be attributed to the small sample size of this case series. Enrolling a larger number of patients may help to obtain significant results through stratification by group. Furthermore, another study limitation is related to the variable characteristics of patients in terms of etiology, duration and size of NK as well as for previous therapies.

In conclusion, this cases series reported promising results for topical insulin, used alone or in combination with DDCL, as demonstrated by the complete healing of NK that was achieved in all cases. Randomized controlled clinical trials are needed in the future to determine the specific contribution of each therapeutic strategy (insulin eye drops and DDCL) for reaching the positive outcomes.

## CRediT authorship contribution statement

**Alessandra Mancini:** Writing – review & editing, Writing – original draft, Validation, Methodology, Investigation, Formal analysis, Data curation, Conceptualization. **Maura Mancini:** Writing – original draft, Validation, Investigation, Data curation. **Andrea Taloni:** Formal analysis, Data curation. **Luca Bifezzi:** Investigation. **Maria Angela Romeo:** Investigation. **Lorenzo Rijillo:** Investigation. **Mario Verdiglione:** Investigation. **Vincenzo Scorcia:** Writing – review & editing, Visualization, Validation, Supervision, Methodology, Investigation, Data curation, Conceptualization. **Pasquale Aragona:** Writing – review & editing, Validation, Supervision. **Giuseppe Giannaccare:** Writing – review & editing, Writing – original draft, Visualization, Validation, Supervision, Methodology, Investigation, Formal analysis, Data curation, Conceptualization.

## Informed consent statement

Written informed consent was obtained by all patients before any study procedure.

## Research Ethics

This study followed the tenets of the 2013 Declaration of Helsinki.

## Data availability statement

Data are available upon reasonable request.

## Funding

None.

## Declaration of competing interest

The authors declare that they have no known competing financial interests or personal relationships that could have appeared to influence the work reported in this paper.
